# Epidural and Non-epidural Analgesia in Patients Undergoing Open Pancreatectomy: a Retrospective Cohort Study

**DOI:** 10.1007/s11605-019-04136-w

**Published:** 2019-02-26

**Authors:** Jesse V. Groen, David E. F. Slotboom, Jaap Vuyk, Chris H. Martini, Albert Dahan, Alexander L. Vahrmeijer, Bert A. Bonsing, J. Sven D. Mieog

**Affiliations:** 1 Department of Surgery, Leiden University Medical Center, Albinusdreef 2, 2300 RC Leiden, The Netherlands; 2 Department of Anesthesiology, Leiden University Medical Center, Leiden, The Netherlands

**Keywords:** Pain, Pancreatectomy, Epidural analgesia, Morphine, Fluid therapy

## Abstract

**Background:**

The use of epidural analgesia (EA) in pancreatic surgery remains under debate. This study compares patients treated with EA versus non-EA after open pancreatectomy in a tertiary referral center.

**Methods:**

All patients undergoing open pancreatectomy from 2013 to 2017 were retrospectively reviewed. (Non-)EA was terminated on postoperative day (POD) 3 or earlier if required.

**Results:**

In total, 190 (72.5%) patients received EA and 72 (27.5%) patients received non-EA (mostly intravenous morphine). EA was terminated prematurely in 32.6% of patients and non-EA in 10.5% of patients. Compared with non-EA patients, EA patients had significantly lower pain scores on POD 0 (1.10 (0–3.00) versus 3.00 (1.67–5.00), *P* < 0.001) and POD 1 (2.00 (0.50–3.41) versus 3.00 (2.00–3.80), *P* = 0.001), though significantly higher pain scores on POD 3 (3.00 (2.00–4.00) versus 2.33 (1.50–4.00), *P* < 0.001) and POD 4 (2.50 (1.50–3.67) versus 2.00 (0.50–3.00), *P* = 0.007). EA patients required more vasoactive medication perioperatively and had higher cumulative fluid balances on POD 1–3. Postoperative complications were similar between groups.

**Conclusions:**

In our cohort, patients with EA experienced significantly lower pain scores in the first PODs compared with non-EA, yet higher pain scores after EA had been terminated. Although EA patients required more vasoactive medication and fluid therapy, the complication rate was similar.

**Electronic supplementary material:**

The online version of this article (10.1007/s11605-019-04136-w) contains supplementary material, which is available to authorized users.

## Introduction

Epidural analgesia (EA) is the current gold standard for perioperative analgesic management in most major abdominal surgeries.^1,2^ However, in patients undergoing pancreatectomy the reported use of EA varies from 10 to 84%.^3–6^ The most used alternative for EA is the patient-controlled analgesia with intravenous morphine (ivPCAM).^3,5,7^

Although some studies reported better postoperative pain control in patients with EA compared to other analgesic management options, detailed reports on pain outcomes after pancreatectomy are sparse.^4,5,8^ In contrast to the generally held belief of the beneficial reported effect of EA on postoperative complications in abdominal surgery,^3,9,10^ recent studies described adverse effects of EA on postoperative complications, number of intensive care unit (ICU) admissions, and length of hospital stay.^4–7^ Furthermore, EA has been associated with perioperative hemodynamic instability and excessive fluid administration, causing early termination of EA and postoperative complications.^4,5,11^

The aim of this study was to compare patients treated with EA versus non-EA (N-EA) regarding the analgesic outcomes in the first 10 postoperative days (PODs) and clinical outcomes after open pancreatectomy in our tertiary referral center.

## Material and Methods

### Study Design and Patient Selection

This retrospective cohort study was approved by the Medical Ethics Committee (G17.059) of the Leiden University Medical Center (LUMC), was registered at www.trialregister.nl (TC 6871), and is reported according to the STROBE criteria.^12^

All consecutive patients undergoing pancreatectomy at the LUMC, a tertiary referral center, from June 2013 through December 2017, were reviewed. Analgesic outcomes are structurally registered in the medical records since June 2013, therefore this period was selected. Only patients undergoing open pancreatectomy were included (initial laparotomy and initial laparoscopic procedure converted to laparotomy).

### Data Collection

Two authors (J.V.G. and D.E.F.S.) performed retrospective data extraction from medical records according to a predefined case report form. Data up to 90 days after surgery or 30 days after discharge were extracted. Extracted data were randomly reviewed by two authors (C.H.M., anesthesiologist and B.A.B., surgeon) for quality control. Variables of interest included (1) patient-related variables: patient characteristics, history of chronic pancreatitis, American Society of Anesthesiologists (ASA)-score, preoperative drug use (opioids, non-steroidal anti-inflammatory drugs, oral anticoagulants), underlying pathology; (2) anesthesia-related variables: type and duration of anesthesia, type and duration of postoperative analgesic treatment, conversion (e.g., EA to ivPCAM or other analgesia), reason for conversion, type of analgesia following EA or ivPCAM, pain scores, use and duration of vasoactive support, cumulative fluid balances; (3) surgery-related variables: type and duration of surgery, blood loss; (4) postoperative variables: duration of admission to the medium care unit (MCU) or ICU, complications related to analgesia treatment, postoperative complications, length of hospital stay, discharge destination, readmission.

### Definitions

The EA group consisted of patients with an epidural catheter during surgery and the N-EA group consisted of patients with all types of analgesia other than EA. The day of surgery was considered as POD 0. Pain scores were measured on an 11-point numerical rating scale (NRS) to assess pain intensity: ranging from 0 (no pain) to 10 (most extreme pain imaginable). A NRS > 4 is an indicator for adjustment of the analgesic regimen and was therefore the cutoff value between acceptable and non-acceptable pain and used for analyses of patients who reported unacceptable pain during PODs 0–10.^13^ Opioids not part of standard EA or ivPCAM infusion (e.g., intramuscular (IM), subcutaneous (SC), or oral (PO)) were considered “supplemental opioids”. The reason for EA termination was classified as “hemodynamic instability” in case perioperative hemodynamic parameters did not improve despite vasoactive medication and fluid therapy. Postoperative pancreatic fistula, postpancreatectomy hemorrhage, bile leakage, delayed gastric emptying, and chyle leak were all classified by the International Study Group of Pancreatic Surgery definitions.^14–18^ For all these complications, grade B and grade C were considered as clinically relevant. The following complications of analgesia were investigated: opioid-induced respiratory depression, infection of puncture sites, postdural puncture headache, and subdural hematoma. The Clavien-Dindo Classification was used to classify overall postoperative complications per patient.^19^

### Analgesic Management

All patients were preoperatively assessed by an anesthesiologist. Based on the preoperative conditions of the patient, type of surgery, and preferences of both patient and physicians (anesthesiologist and surgeon), a shared decision was made regarding the type of analgesic treatment (i.e., EA or N-EA). None of the involved anesthesiologists and surgeons refused to use either EA or N-EA.

Perioperative hemodynamic therapy was goal-directed according to local protocol: focused on maintaining a mean arterial pressure > 55 mmHg and a urinary output of > 0.5 mL/kg/h and preventing excessive fluid administration.

EA and N-EA treatments were applied according to local protocol. In the case of EA, the epidural catheter was inserted preoperatively at level Th6-Th10. EA patients received 0.2% ropivacaine combined with 0.75 μg/mL sufentanil. The background continuous infusion rate was 4–8 mL/h. If needed, patients could manually administer an additional bolus (2 mL, lockout 20 min). In addition, patients received 1 g acetaminophen (paracetamol) 4 times daily. Because of sterility considerations, EA was terminated 72 h after surgery (i.e., on POD 3). Thereafter, patients received a combination of acetaminophen and nurse-administered IM/SC/PO opioids (in absence of contraindications) depending on NRS scores.

Patients with N-EA generally received intravenous (IV) morphine bolus doses postoperatively to reduce pain scores ≤ 4, followed by ivPCAM. IvPCAM included a background infusion rate of 0.5 mg/h. In addition, the patients could administer a 1 mg bolus at a 5 min interval with a maximum dosage of 28 mg per 4 h. Furthermore, patients received 1 g acetaminophen 4 times daily. IvPCAM was terminated 72 h after surgery (i.e., on POD 3). Thereafter, patients continued to receive acetaminophen now combined with nurse-administered IM/SC/PO opioids (in absence of contraindications) depending on pain scores. The Acute Pain Service^20^ was responsible for analgesic management for the duration of EA or ivPCAM. The Acute Pain Service visited the patients twice daily to evaluate and modify analgesic management if needed. Together with the nursing staff, they were responsible for measuring pain scores (on the NRS) at least three times a day according to national protocol.^21^

### Outcomes

The primary outcome of this study was the mean pain scores and percentage of patients who reported unacceptable pain per POD. The secondary outcomes were the details of analgesic treatment (percentage, timing and reason of premature termination of initial analgesic technique, and use of supplemental opioids), perioperative hemodynamics (use of vasoactive medication and cumulative fluid balances), and the postoperative outcomes (postoperative complications, mortality and length of hospital stay).

### Statistical Analysis

Continuous variables are presented as mean (standard deviation) or median (interquartile range) and compared by unpaired *t* tests or Mann-Whitney U tests, depending on their distribution. Categorical variables are presented as numbers (percentages) and compared by chi-square or Fisher’s exact tests. For analyses of pain scores, we calculated the mean NRS per patient per POD and identified patients who reported unacceptable pain (pain score > 4) at least once per POD. Because the mean pain scores are not normally distributed, values are presented as median (IQR). Cumulative fluid balances were calculated per patient by adding up fluid balances of preceding days and the POD of interest. Main analyses were based on the comparison of patients with EA versus patients with N-EA. Subgroup analyses were performed with patients who completed the first three PODs with their initial analgesic technique (i.e., successful EA versus successful ivPCAM). For statistical analyses, SPSS Inc. for Windows (version 23.0) was used. *P* values < 0.05 were considered significant.

## Results

### Patient Characteristics and Details of Analgesic Treatment

In total, the study cohort consisted of 262 patients: 190 (72.5%) patients in the EA group and 72 (27.5%) in the N-EA group (Table 1). Both groups were comparable for patient and intraoperative characteristics. However, in the N-EA group, ASA-score, the use of oral anticoagulants and blood loss was higher. In the N-EA group, 64 patients received ivPCAM, six patients received nurse-administered IM/SC/PO opioids, and two patients received a continuous infusion of sufentanil after surgery. Reasons not to use EA were medical contraindication (*N =* 28), preoperative failure of placement (*N =* 20), physicians’ preference (*N =* 15), and patients’ preference (*N =* 9). Type of resection did not differ between groups (*P =* 0.161).

**Table 1:** Patient and intraoperative characteristics

	Type of analgesia		
	EA	N-EA^*^	*P*
	(*N* = 190; 72.5%)	(*N* = 72; 27.5%)	
Sex, *n* (%)			0.688
Male	95 (50.0)	38 (52.8)	
Female	95 (50.0)	34 (47.2)	
Age, mean (SD)	62 (13)	64 (11)	0.395
BMI, mean (SD)	25.3 (4.4)	26.5 (5.2)	0.064
History of chronic pancreatitis *n* (%)	21 (11.1)	6 (8.3)	0.518
Preoperative opioid use, *n* (%)	15 (7.9)	10 (13.9)	0.140
Preoperative NSAID use, *n* (%)	31 (16.3)	9 (12.5)	0.443
Preoperative OAC use, *n* (%)	8 (4.2)	9 (12.5)	0.015
ASA-score, *n* (%)			0.024
I	27 (14.2)	6 (8.3)	
II	133 (70.0)	44 (61.1)	
III	30 (15.8)	21 (29.2)	
IV	0	1 (1.4)	
Reason no EA, *n* (%)			–
Medical contraindication	–	28 (38.9)	
Preoperative placement failure	–	20 (27.8)	
Physicians’ preference	–	15 (20.8)	
Patients’ preference	–	9 (12.5)	
Type of anesthesia^†^, *n* (%)			0.988
TIVA (propofol)	172 (91.5)	65 (91.5)	
Sevoflurane	16 (8.5)	6 (8.5)	
Type of resection, *n* (%)			0.161
PPPD/classic whipple	142 (74.7)	44 (61.1)	
Total pancreatectomy	12 (6.3)	5 (6.9)	
Distal pancreatectomy	33 (17.4)	20 (27.8)	
Central pancreatectomy	1 (0.5)	2 (2.8)	
Enucleation	2 (1.1)	1 (1.4)	
Laparotomy after conversion^‡^, *n* (%)	4 (2.1)	8 (11.1)	< 0.001
Blood loss, median (IQR)	800 (450–1225)	1100 (750–1750)	< 0.001
Operation time (min), mean (SD)	259 (75)	261 (75)	0.837
Vascular resection^§^, *n* (%)	30 (15.8)	6 (8.3)	0.118
Multi-visceral resection^¶^, *n* (%)	58 (30.5)	24 (33.3)	0.662
Underlying pathology, *n* (%)			0.213
Adenocarcinoma	134 (70.5)	45 (62.5)	
Other	56 (29.5)	27 (37.5)	

*(N-)EA* (non-)epidural, *SD* standard deviation, *BMI* Body Mass Index *IQR* interquartile range *NSIAD* non-steroidal anti-inflammatory drugs, *OAC* oral anticoagulants,*ASA* American Society of Anesthesiologists, *TIVA* total intravenous anesthesia, *PPPD* pylorus-preserving pancreaticoduodenectomy

^*^Included patients with intravenous patient-controlled analgesia with morphine, NSIADs, oral/subcutaneous opioids only, and sufentanil perfusor

^†^Missing data: two patients in the EA group, one patient in N-EA group

^‡^Considered as conversion during a laparoscopic intended resection (not diagnostic laparoscopy)

^§^Included wedge–and segmental resection of the superior mesenteric vein, portal vein, or hepatic artery

^¶^Included resections of spleen, liver, stomach, small bowel, colon, adrenals, and kidney

**Fig. 1:**
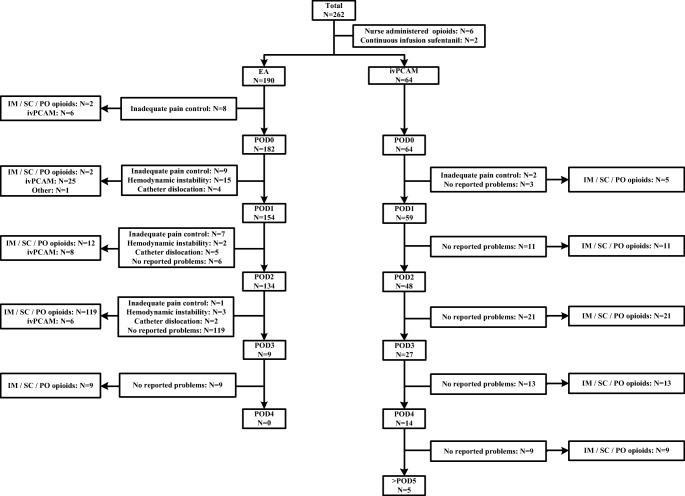
Flow chart of the use of EA and ivPCAM per POD

Initial analgesia was terminated on POD 3 without reported problems (according to protocol) in 119 (62.6%) patients with EA and 21 (32.8%) patients with ivPCAM (Fig. 1). In 62 (32.6%) patients EA was terminated prematurely due to inadequate pain control (*N* = 25), hemodynamic instability (*N* = 20), catheter dislocation (*N* = 11), and without reported problems (*N* = 6). In the patients with prematurely terminated EA, 41 patients received ivPCAM following EA (*N* = 6 on POD 0; *N* = 25 on POD 1; *N* = 8 on POD 2; and *N* = 2 on POD 3). In addition, four patients received ivPCAM after termination of EA according to protocol. IvPCAM was terminated prematurely in 16 (10.5%) patients, due to inadequate pain control (*N* = 2) and without reported problems (*N* = 14). All ivPCAM patients received nurse-administered IM/SC/PO opioids after termination of ivPCAM.

### Primary Outcome

**Fig. 2:**
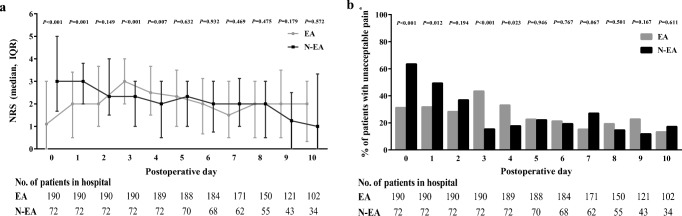
**a** Median (IQR) of mean pain score per POD and **b** Patients with unacceptable pain per POD. ^*^Patients who reported a pain score > 4 at least once per POD

Patients in the EA group had statistically significant lower mean pain scores on POD 0 (1.10 (0–3.00) versus 3.00 (1.67–5.00)) and POD 1 (2.00 (0.50–3.41) versus 3.00 (2.00–3.80)), whereas they experienced statistically significantly higher mean pain scores on POD 3 (3.00 (2.00–4.00) versus 2.33 (1.50–4.00)) and POD 4 (2.50 (1.50–3.67) versus 2.00 (0.50–3.00); Fig. 2a). From POD 5 forward there were no significant differences between groups.

The EA group reported unacceptable pain (pain scores > 4) significantly less often on POD 0 (31.2% versus 63.5%, *P* < 0.001 Fig. 2b); and POD 1 (31.7% versus 49.3%, *P* = 0.012). Conversely, the EA-group reported unacceptable pain significantly more often on POD 3 (43.4% versus 15.4%, *P* < 0.001) and POD 4 (33.1% versus 17.7%, *P* = 0.023). From POD 5 forward there were no significant differences between groups.

Subgroup analyses showed that EA patients who completed POD 0 (*N* = 182), POD 1 (*N* = 154) and POD 2 (*N* = 134) experienced significantly lower mean pain scores and less unacceptable pain per POD compared to patients with N-EA (Fig. S1a–b).

### Secondary Outcomes

#### Use of Supplemental Opioids

**Fig. 3:**
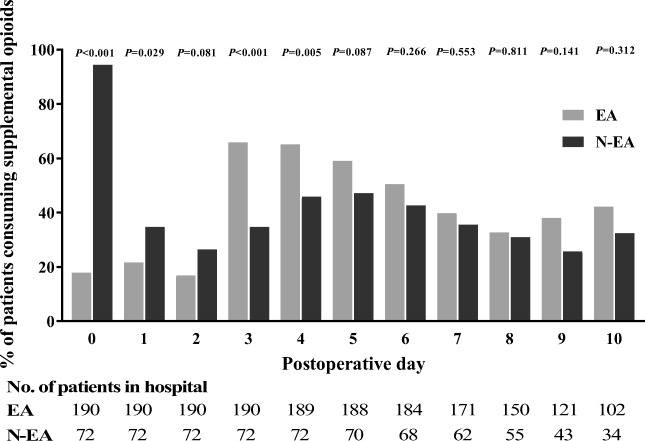
Supplemental opioid consumption per POD

More N-EA patients required supplemental opioids to treat their pain on PODs 0–1 (Fig. 3). In contrast, on PODs 3–4 significantly more EA patients required supplemental opioids. From POD 5 forward there were no significant differences between groups.

#### Perioperative Hemodynamics

The EA group received more vasoactive medication perioperatively, demonstrated by a significantly higher total dosage of noradrenaline, over a longer period, and with a higher maximum infusion rate (Table 2). Also, the total dosages of phenylephrine and ephedrine were significantly higher in the EA group.

**Table 2:** Perioperative characteristics

	Type of analgesia		
	EA	N-EA^*^	*P*
	(*N* = 190; 72.5%)	(*N* = 72; 27.5%)	
Duration of anesthesia (min), median (IQR)	301 (257–355)	308 (260–349)	0.740
Intraoperative need of vasoactive medication, *n* (%)	186 (97.9)	63 (87.5)	< 0.001
Noradrenaline, *n* (%)	152 (80.0)	49 (68.1)	0.041
Phenylephrine, *n* (%)	145 (76.3)	47 (65.3)	0.071
Ephedrine, *n* (%)	125 (65.8)	29 (40.3)	< 0.001
Postoperative MC/ICU admission, *n* (%)	168 (88.4)	58 (80.6)	0.099
Duration of postoperative MC/ICU admission (min), median (IQR)	1174 (1055–1325)	1185 (900–1293)	0.157
Postoperative MC/ICU need of vasoactive medication, *n* (%)	140 (73.7)	31 (43.1)	< 0.001
Noradrenaline, *n* (%)	131 (68.9)	29 (40.3)	< 0.001
Phenylephrine, *n* (%)	19 (10.0)	6 (8.3)	0.682
Ephedrine, *n* (%)	3 (1.6)	0	0.284
Total dose of noradrenaline (mg), median (IQR)	2.08 (0.45–4.58)	0.64 (0–6.00)	< 0.001
Duration of infusion noradrenaline (min), median (IQR)	790 (153–1240)	181 (0–402)	< 0.001
Maximum infusion rate noradrenaline μg/kg/min, median (IQR)	0.10 (0.04–0.15)	0.07 (0–0.11)	0.025
Total dose of phenylephrine (μg), median (IQR)	500 (100–1200)	200 (0–700)	0.009
Total dose of ephedrine (mg), median (IQR)	10.0 (0–17.5)	0 (0–10.0)	< 0.001

*Min* minutes, *IQR* interquartile range, *MC/ICU* medium care/intensive care unit, *mg* milligram, *μg* microgram, *kg* kilogram

^*^Included patients with intravenous patient-controlled analgesia with morphine, NSIADs, oral/subcutaneous opioids only, sufentanil perfusor

**Fig. 4:**
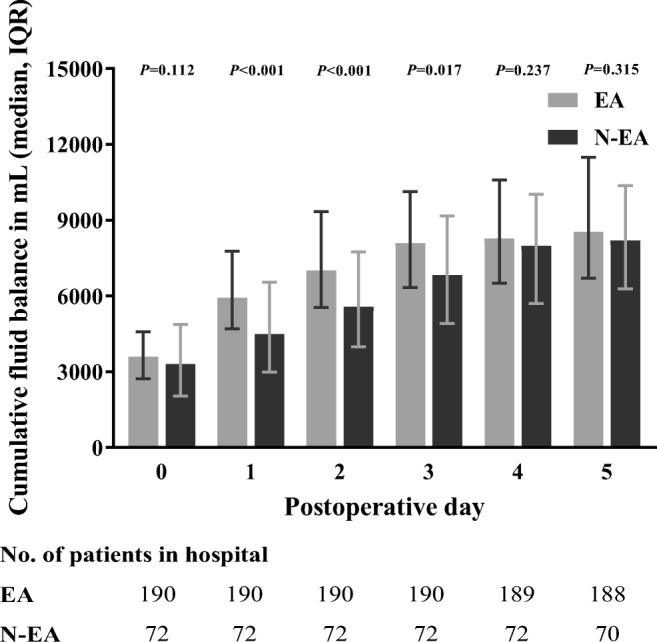
Median (IQR) cumulative fluid balances (mL) per POD

Both groups had a similar cumulative fluid balance on POD 0 (Fig. 4). While on PODs 1–3, the cumulative fluid balance was significantly higher in the EA group (POD1 5930 (4693–7765) mL versus 4485 (2982–6548) mL, *P* < 0.001). From POD 4 forward, there were no significant differences between groups.

#### Postoperative Outcomes

There were no differences between groups regarding postoperative complications and Clavien-Dindo classification (Table 3). In the EA group, three patients had an opioid-induced respiratory depression (EA was already terminated) on the surgical ward which was treated with naloxone without further clinical consequence. No other complications related to analgesia occurred. In total, 7 (3.7%) patients in the EA group and one (1.4%) patient in the N-EA group deceased within 90-days after surgery (*P =* 0.335). In all deceased patients, the cause was not related to the type of analgesia. The length of hospital stay did not differ between the two groups.

**Table 3:** Postoperative outcomes

	Type of analgesia		
	EA	N-EA^*^	*P*
	(*N* = 190; 72.5%)	(*N* = 72; 27.5%)	
CR-POPF^†^, *n* (%)	29 (15.3)	9 (12.5)	0.571
CR-PPH^†^, *n* (%)	37 (19.5)	18 (25.0)	0.327
CR-BL^†^, *n* (%)	10 (5.3)	2 (2.8)	0.390
CR-DGE^†^, *n* (%)	43 (22.6)	18 (25.0)	0.686
CR-CL^†^, *n* (%)	5 (2.6)	3 (4.2)	0.519
Woundinfection, *n* (%)	12 (6.3)	8 (6.9)	0.854
Pneumonia, *n* (%)	12 (6.3)	4 (5.6)	0.819
Intraabdominal abscess, *n* (%)	26 (13.7)	15 (20.8)	0.155
Complications of analgesia, *n* (%)	3 (1.6)	0	0.284
Reintervention, *n* (%)	49 (25.8)	16 (22.2)	0.551
Relaparotomy	21 (11.1)	7 (9.7)	0.756
Radiological intervention	42 (22.1)	14 (19.4)	0.639
ICU admission, *n* (%)	31 (16.3)	9 (12.5)	0.443
Length of ICU admission^‡^, median (IQR)	3 (1–22)	2 (1–7)	0.564
Clavien-Dindo classification^§^, *n* (%)			0.419
No complications	55 (28.9)	26 (36.1)	
I–II	77 (40.5)	29 (40.3)	
III–V	58 (30.5)	17 (23.6)	
Ninety-day mortality, *n* (%)	7 (3.7)	1 (1.4)	0.335
Length of hospital stay (days), median (IQR)	10 (8–14)	9 (8–15)	0.741
Discharge destination, *n* (%)			0.354
Home	101 (54.6)	33 (46.5)	
Home + additional care	53 (28.6)	21 (29.6)	
Rehabilitation facility	31 (16.8)	17 (23.9)	
Readmission, *n* (%)	30 (16.3)	16 (22.5)	0.246

*CR* clinically relevant*, POPF* postoperative pancreatic fistula, *PPH* postpancreatectomy hemorrhage, *BL* bile leakage, *DGE* delayed gastric emptying, *CL* chyle leakage *ICU* intensive care unit, *IQR* interquartile range

^*^Included patients with intravenous patient-controlled analgesia with morphine, NSIADs, IM/SC/PO opioids only, amd sufentanil perfusor

^†^As defined and classified by the International Study Group Pancreatic Surgery^15–19^

^‡^In case of ICU admission

^§^Classified according the Clavien-Dindo classification^20^

## Discussion

This study showed EA was used in 72.5% of patients undergoing open pancreatectomy. There were several important outcomes of the comparison between EA and N-EA patients: (1) initial analgesia was prematurely converted to another form of analgesia in 32.6% of EA patients versus 10.5% of N-EA patients; (2) EA patients had lower mean pain scores and fewer reported unacceptable pain on PODs 0–1. However, termination of EA led to higher mean pain scores and more patients reported unacceptable pain on POD 3–4, which led to the need for the liberal administration of supplemental opioids; (3) the EA group received more vasoactive medication perioperatively and also cumulative fluid balances were significantly higher on PODs 1–3; (4) postoperative complications and length of hospital stay were similar between both groups. Previous studies comparing EA with N-EA reported mixed results regarding pain scores and postoperative complications in relatively small cohorts of patients undergoing pancreatoduodenectomy (PD) and major hepatopancreatobiliary (HPB) surgery.^4,5,7,22,23^ A recent randomized controlled trial in patients undergoing major HPB surgery showed improved pain control and similar postoperative outcomes between the EA and ivPCAM group, although only 3% of included patients underwent pancreatectomy.^24^ Therefore, our large cohort study of solely patients undergoing pancreatectomy provides insight into the effects of analgesic technique. The forthcoming results of a randomized controlled trial comparing EA versus ivPCAM in patients undergoing PD could clarify the influence of analgesic technique on postoperative outcomes.^25^

Possible solutions for the higher pain scores after termination of EA might be extending the EA phase or by a preemptive and more strict analgesic treatment (opioid or non-opioid) during the transition from EA to other analgesia. A prolonged EA phase (PODs 4–6) is already implemented in some other centers.^5,6,22,24,26^ Unlike our study, these studies did not report results after termination of EA. Therefore, it is unclear whether extending the EA phase after POD 3 (and delaying the transition from EA to other analgesia) would lead to lower pain scores and less use of opioids. Moreover, previous studies and our study showed the association between EA and perioperative hemodynamic instability, leading to early termination in 7–41% of EA (10.5% in our study).^5–8,26,27^ The higher cumulative fluid balances on PODs 1–3 in the EA group can be explained by the switch from vasoactive medication at the MC/IC to fluid therapy on the surgical ward to ensure adequate hemodynamic status. We hypothesize that excessive fluid therapy on the surgical ward is needed as long as the EA phase is prolonged. Therefore, we suggest not to extend the EA phase but to apply a multimodal analgesic regimen that covers the increase in pain scores upon EA termination.

The high rate of premature termination of EA and worse pain control with ivPCAM implicate that a new alternative for postoperative analgesia is needed. Alternatives for postoperative analgesia have been investigated in previous studies. One study reported results of continuous wound infiltration compared to EA showing lower pain scores, less opioid side-effects, and less use of vasoactive medication after HPB surgery.^28^ A possible disadvantage is that the use of multiple wound catheters and pumps might impede early mobilization of the patient. Another study showed that pain scores after subcostal transversus abdominis plane catheters were comparable with EA in upper abdominal surgery.^29^ However, the catheters needed resiting in 45% of patients. Sublingual sufentanil tablets (SST) have been investigated and showed promising pain scores and safety parameters after open abdominal surgery.^30^ SST are rapidly absorbed, causing a minimal delay in pain relief, and because peak concentrations are low typical opioid side effect occur less frequent.^31^ The occurrence of other side effects (e.g., headaches and hypotension) is comparable with other forms of opioid treatment.^32^ We started an investigator-initiated, multicenter, randomized controlled trial to compare SST and EA in patients undergoing PD (www.trialregister.nl: TC 7318).

Our study has several limitations. The registration of side effects (e.g., nausea, pruritus) of analgesia was not reported in a standardized manner, which did not allow comparisons between groups. Our data indicate that the shared decision (by the anesthesiologist, surgeon and patient) to determine the postoperative analgesic technique is partly based on patient characteristics: the N-EA group had a higher ASA-score and more oral anticoagulant users. It may well be that comparison of outcomes between EA and N-EA patients are not just related to the analgesia technique but also to patient selection. We performed sensitivity analyses with patients undergoing PD (70.2% of patients) which showed similar results regarding all outcomes (data not shown). Nevertheless, in contrast to previous studies, this study presents a large cohort of open pancreatectomies with detailed data of analgesic management in the first 10 PODs and postoperative outcomes.

## Conclusion

In our cohort, patients receiving EA after open pancreatectomy had significantly lower pain scores in the first PODs compared with non-EA, yet higher pain scores after EA was terminated. Although EA patients required more vasoactive medication and fluid therapy, postoperative complications were similar between groups.

## Electronic supplementary material

Figure S1 Subgroup analysis of in situ analgesia: (**a**) Median (IQR) of mean pain score per POD & (**b**) Patients with unaccpetable pain per POD. * Patients who reported a pain score >4 at least once per POD. (PDF 44.3 kb)
