# Supplementary material for: Epidural and Non-epidural Analgesia in Patients Undergoing Open Pancreatectomy: a Retrospective Cohort Study
Source: J Gastrointest Surg. 2019 Feb 26;23(12):2439–48. doi: 10.1007/s11605-019-04136-w (PMC6877489; doi:10.1007/s11605-019-04136-w)
Supplement: Supplementary file 1 — Subgroup analysis of in situ analgesia: (a) Median (IQR) of mean pain score per POD & (b) Patients with unaccpetable pain per POD. * Patients who reported a pain score >4 at least once per POD. (PDF 44.3 kb) [file 11605_2019_4136_MOESM1_ESM.pdf]

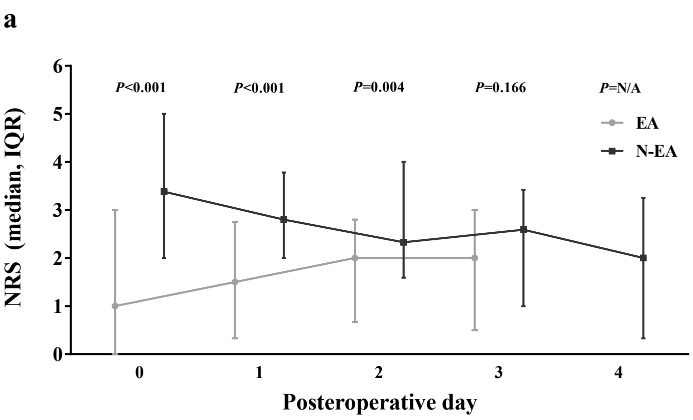


| No. of patients with *in situ* EA or ivPCAM | | | | | |
|---|---|---|---|---|---|
| EA | 182 | 154 | 134 | 9 | - |
| ivPCAM | 64 | 59 | 48 | 27 | 14 |

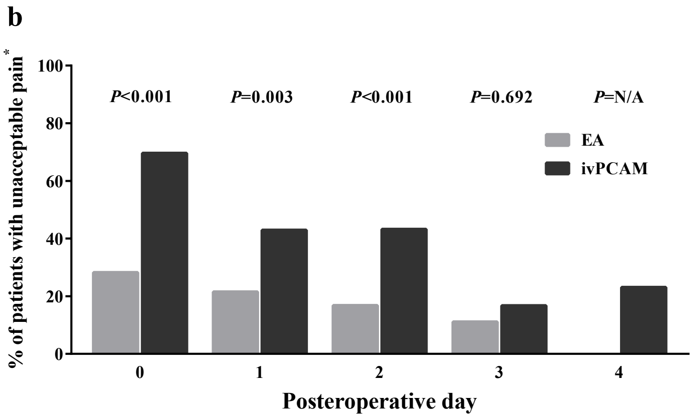


| No. of patients with *in situ* EA or ivPCAM | | | | | |
|---|---|---|---|---|---|
| EA | 182 | 154 | 134 | 9 | - |
| ivPCAM | 64 | 59 | 48 | 27 | 14 |
